# The impact of Healthy Conversation Skills training on health professionals’ barriers to having behaviour change conversations: a pre-post survey using the Theoretical Domains Framework

**DOI:** 10.1186/s12913-021-06893-4

**Published:** 2021-08-27

**Authors:** Jenna L. Hollis, Lucy Kocanda, Kirsty Seward, Clare Collins, Belinda Tully, Mandy Hunter, Maralyn Foureur, Wendy Lawrence, Lesley MacDonald-Wicks, Tracy Schumacher

**Affiliations:** 1 Hunter New England Population Health, Hunter New England Local Health District, Newcastle, New South Wales Australia; 2 School of Medicine and Public Health, The University of Newcastle, Newcastle, New South Wales Australia; 3 Hunter Medical Research Institute, Newcastle, New South Wales Australia; 4 Priority Research Centre for Health Behaviour, The University of Newcastle, Newcastle, New South Wales Australia; 5 Priority Research Centre for Physical Activity and Nutrition, The University of Newcastle, Newcastle, New South Wales Australia; 6 Department of Rural Health, University of Newcastle, Tamworth, New South Wales Australia; 7 School of Health Sciences, The University of Newcastle, Newcastle, New South Wales Australia; 8 Hunter New England Local Health District Nursing and Midwifery Services, Newcastle, New South Wales Australia; 9 School of Nursing and Midwifery, The University of Newcastle, Newcastle, New South Wales Australia; 10 Nursing and Midwifery Research Centre, Hunter New England Health, Newcastle, New South Wales Australia; 11 Medical Research Council Lifecourse Epidemiology Centre, University of Southampton, Southampton, UK; 12 NIHR, Southampton Biomedical Research Centre, University Hospital Southampton NHS Foundation Trust, Southampton, UK

**Keywords:** Behaviour change theory, Communication skills, Health promotion, Healthy Conversation Skills, Theoretical Domains Framework, Workforce development

## Abstract

**Background:**

Changing people’s behaviour by giving advice and instruction, as traditionally provided in healthcare consultations, is usually ineffective. Healthy Conversation Skills (HCS) training enhances health professionals’ communication skills and ability to empower and motivate people in health behaviour change. Guided by the Theoretical Domains Framework (TDF), this study examined the impact of HCS training on health professional barriers to conducting behaviour change conversations in both clinical and non-clinical settings. Secondary aims were to i) identify health professionals’ barriers to having behaviour change conversations, and explore the ii) effect of HCS training on health professionals’ competence and attitudes to adopting HCS, iii) feasibility, acceptability and appropriateness of using HCS in their clinical and non-clinical roles, and iv) acceptability and quality of HCS training.

**Methods:**

HCS training was conducted in October-November 2019 and February 2020. Pre-training (T1), post-training (T2) and follow-up (T3; 6-10 weeks post-training) surveys collected data on demographics and changes in competence, confidence, importance and usefulness (10-point Likert scale, where 10 = highest score) of conducting behaviour change conversations. Validated items assessing barriers to having these conversations were based on eight TDF domains. Post-training acceptability and quality of training was assessed. Data were summarised using descriptive statistics, and differences between TDF domain scores at the specific time points were analysed using Wilcoxon matched-pairs signed-rank tests.

**Results:**

Sixty-four participants consented to complete surveys (97% women; 16% identified as Aboriginal), with 37 employed in clinical settings and 27 in non-clinical settings. The training improved scores for the TDF domains of skills (T1: median (interquartile range) = 4.7(3.3-5.3); T3 = 5.7(5.3-6.0), *p* < 0.01), belief about capabilities (T1 = 4.7(3.3-6.0); T3 = 5.7(5.0-6.0), *p <* 0.01), and goals (T1 = 4.3(3.7-5.0); T3 = 4.7(4.3-5.3), p < 0.01) at follow-up. Competence in using ‘open discovery questions’ increased post-training (T1 = 25% of responses; T2 = 96% of responses; T3 = 87% of responses, *p* < 0.001), as did participants’ confidence for having behaviour change conversations (T1 = 6.0(4.7-7.6); T2 = 8.1(7.1-8.8), *p <* 0.001), including an increased confidence in having behaviour change conversations with Aboriginal clients (T1 = 5.0(2.7-6.3); T2 = 7.6(6.4-8.3), *p <* 0.001).

**Conclusions:**

Provision of additional support strategies to address intentions; memory, attention and decision processes; and behavioural regulation may enhance adoption and maintenance of HCS in routine practice. Wider implementation of HCS training could be an effective strategy to building capacity and support health professionals to use a person-centred, opportunistic approach to health behaviour change.

**Supplementary Information:**

The online version contains supplementary material available at 10.1186/s12913-021-06893-4.

## Background

Behavioural risk factors such as smoking, poor diet, excess alcohol consumption and physical inactivity are key risk factors to target for prevention and treatment of obesity and chronic disease [1]. The demand for health promotion and behaviour change interventions continues to increase across primary, secondary and tertiary healthcare settings [2, 3]. However, health professionals providing people with knowledge via didactic communication processes focussed on advice-giving and instruction, as used in the traditional healthcare medical model, is usually insufficient to change individual’s behaviour. Patients and clients must also feel motivated and able to change [4, 5]. Person-centred counselling approaches are characterised by exploratory conversations through which health professionals acknowledge the complexities of behaviour change, attempt to understand the world of the person and the context of their behaviour, and support them to plan their own solutions [6]. As such, people are involved in a process of empowerment by which they take control of their behaviours, and increase their sense of self-efficacy [6].

**Fig. 1:**
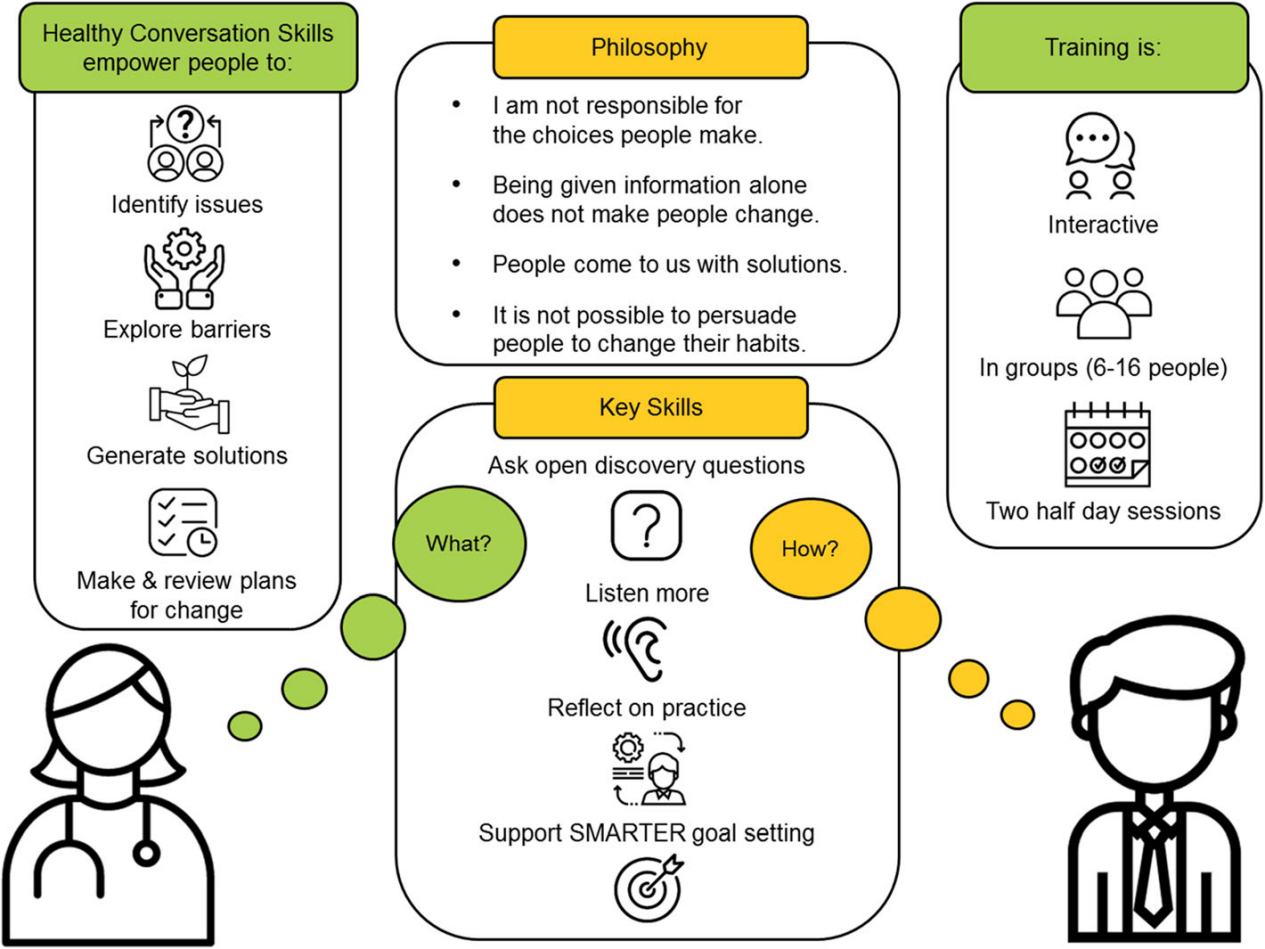
A description of Healthy Conversation Skills philosophy, skills and training delivery

Healthy Conversation Skills (HCS) adopts this empowering, person-centred approach to health behaviour change (Fig. 1), and is advocated as an effective approach to support individuals in behaviour changes to address chronic disease risk factors [7]. HCS was developed originally to support frontline health professionals in effectively empowering pregnant women in Southampton, United Kingdom to adopt behaviours to improve their dietary intakes [8]. Health professionals are trained in communication skills to maximise the benefit from conversations with clients that support them to find their own solutions and identify first steps to change. Since the first delivery and evaluation of HCS, the training has been further refined to meet the needs of a range of health professionals working across both clinical and non-clinical settings, with different populations and in multiple global contexts [9–11]. The skills are practical, straight forward to learn and can be used opportunistically by practitioners in any time frame [9–11]. HCS is based on Social Cognitive Theory [12] and training delivery is underpinned by the Taxonomy of Behaviour Change Techniques [13] (see Additional file 1). HCS-trained practitioners have demonstrated improved confidence and competence in supporting individual’s in behaviour change and demonstrated continued use of these skills up to 1 year post-training [10, 14, 15]. Studies have shown that patient groups prefer HCS communication, and it leads to productive behaviour change conversations [9, 11]. A Canadian study showed that pregnant women in the intervention arm were more satisfied with care provided by a research dietitian trained to have healthy conversations with them than those in the control arm where the dietitian delivered standard advice-giving care [9]. This study and a UK trial found that pregnant participants who experienced healthy conversations, set more behaviour change goals and made more positive changes to their diet and/or physical activity [9, 11].

Health professionals are ideally placed to support health behaviour change using HCS as they are a trusted source of health support [16] and have frequent client contact which provides many opportunities to have empowering conversations [17]. However, health professionals working across a range of settings have described multiple barriers to engaging in behaviour change conversations, including a lack of skills and/or confidence [18, 19]. Other barriers include concern about potential negative impact on client-professional relationship, a belief that it is not appropriate within their health professional role, and environmental factors such as having insufficient time to use a person-centred approach [18–20]. An evaluation of how HCS training ameliorates health professionals’ barriers to having behaviour change conversations has not been reported.

A better understanding of which health professional barriers and enablers to supporting an individual’s behaviour change that are currently not addressed through HCS training can help refine future training and inform additional support strategies to enhance adoption and maintenance of HCS in routine practice [21, 22].

Outcomes, such as feasibility, acceptability and appropriateness, can serve as indicators of implementation processes in formative research and of the preconditions needed to attain desired health service delivery [23]. A randomised controlled trial (RCT) of HCS with pregnant women in the UK has shown that research nurses/midwives and participants find the conversations feasible, acceptable and useful within consultations [11]. Exploring the implementation of HCS with health professionals working in different disciplines, in other countries and employed in varied health service systems, is needed to understand and synthesise cross-disciplinary outcomes and experiences of HCS. This will ensure the training meets the needs of diverse health professional groups and supports the delivery of behaviour change interventions at scale. The primary aim of the current study was to evaluate the impact of HCS training on health professionals’ barriers to having behaviour change conversations in both clinical and non-clinical settings, using the Theoretical Domains Framework (TDF) to identify mechanism(s) of action. Secondary aims were to i) identify health professionals’ barriers to having behaviour change conversations, and explore the ii) effect of HCS training on health professionals’ competence and attitudes to adopting HCS, iii) feasibility, acceptability and appropriateness of using HCS in their clinical and non-clinical role, and iv) acceptability and quality of HCS training.

## Methods

### Design

The study incorporated a prospective pre-post survey design to evaluate the impact of the training, participants’ experiences of attending training and of applying HCS in their professional role. The study, from conception and inclusive of all processes, underwent review to ensure the cultural inclusion, appropriateness and safety for Aboriginal health professionals attending the training and for Aboriginal peoples/community receiving care from health professionals trained in HCS. The study was approved by the Hunter New England Human Research Ethics Committee (2019/ETH13158), and the University of Newcastle Human Research Ethics Committee (H-2019-0409)). All methods were carried out in accordance with relevant guidelines and regulations.

### Participants and procedure

A convenience sample of 77 health professionals working in New South Wales public, private and not-for-profit health services, including Aboriginal health professionals, and university teaching and research staff, were invited to participate in the HCS training and to complete the surveys. Members of the project team identified and invited (via email) potential participants through their existing networks based on individuals’ interest in and ability to attend the training, and capacity to embed HCS within their job role. Information statements were emailed to participants prior to attending training and provided again at the beginning of each survey and the telephone interview to inform them about the study, and consent was implied by completing each survey.

### Healthy Conversation Skills training

HCS training consisted of four hours on two consecutive days (eight hours in total) at two locations in Newcastle and Tamworth (Australia) in October-November 2019 and February 2020. Five training courses were conducted, with between six and sixteen participants in each course. Participants were offered on-going support through a telephone call from a trainer within 6-10 weeks post-training to discuss how HCS were being implemented in practice. Training was delivered by four HCS trainers (one global HCS lead, and three new trainers), with one to two trainers delivering each course. New trainers co-delivered the course with the global HCS lead trainer.

HCS training is based on Social Cognitive Theory [12], recognising the important role of self-efficacy in determining whether an individual undertakes an action or not. By building self-efficacy for having behaviour change conversations, it is envisaged that these will be more likely to occur. In turn by having healthy conversations, practitioners can enhance their clients’ self-efficacy towards achieving their desired goals. HCS training uses an interactive, participatory approach to learning, with the trainer modelling the HCS philosophy and skills throughout. There is no use of technology, participants are discouraged from taking notes and encouraged to fully engage with the experiential style of training. HCS training is built on the pedagogy of active learning.

The training is underpinned by the Taxonomy of Behaviour Change Techniques [13], in order to maximise the likelihood of the adoption of the skills (see Additional file 1). For example, the training provides opportunities for participants to reflect on discrepancies between their current communication style and desired HCS communication style, demonstrates a ‘real’ healthy conversation, supports participants to practise the skills, and sets graded tasks to progressively increase skill level. The use of group work to provide social support and feedback, as well as sharing experiences and practising with each other is fundamental to this approach to learning.

### Data collection procedures and measures

Evaluation data were collected at three time points. The pre-training (T1; 52 questions) and post-training (T2; 59 questions) surveys were conducted immediately prior to and at the conclusion of training to maximise participant response rates and increase the validity of the measures by minimising recall bias and controlling for the influence of confounding factors (e.g. discussions with colleagues, and engaging in additional training). The follow-up survey (T3; 55 questions) was conducted 6-10 weeks post-training to evaluate impact of the training once participants had the opportunity to use their new skills within the workplace. A semi-structured interview via a telephone call was also offered to all participants at 6-10 weeks post-training. Participants were offered the option to complete the surveys online or via paper copies. Each survey was estimated to take 15 min to complete. The telephone interview was estimated to take 10 to 20 min.

Demographic and health profession data including participant sex, Aboriginal or Torres Strait Islander origin, employer organisation, position, type of health service delivery/research/teaching, years of professional experience, and purpose of attending training were collected in the pre-training survey. Survey items assessing potential barriers and enablers to having behaviour change conversations with clients were developed from the TDF. A behaviour change conversation was defined as *‘a talk or discussion with a client/individual about changing their actions or habits to improve their health and wellbeing’.* The TDF is an integrative, validated framework of 33 behaviour change theories developed using a consensus approach and enables the mapping of barriers and enablers to 12-14 specific behavioural domains [24, 25]. It has previously been used to theoretically assess the barriers and enablers to the adoption of a wide range of healthcare initiatives, and can be used to understand the mechanisms of action of an intervention [22, 24]. Previous studies measuring the psychometric properties of surveys applying the TDF have demonstrated good content and face validity and internal consistency [24, 26, 27]. Eight of the domains of the TDF survey developed by Huijg et al. [26] were included in the survey: skills; social/professional role and identify; belief about capabilities; belief about consequences; intentions; goals; memory; attention and decision processes; and behaviour regulation (action planning) (see Additional file 2). The selection of these domains was informed by previous surveys with health professionals [18, 28], systematic reviews examining barriers and enablers to implementing behaviour change interventions in a number of different health care settings [29, 30], and consultation with health service and university teaching partners. TDF survey items primarily assessed changes in potential barriers and facilitators using a 7-point Likert scale (from strongly disagree/not strong at all/never (1) to strongly agree/very strong/always (7)). For one TDF survey item (intentions domain) participants reported the score out of 10, which was scaled to a maximum score of seven for the analysis.

Changes in competence, confidence, importance and usefulness of having conversations with clients about behaviour change were measured in each of the pre-, post- and follow-up surveys. To measure change in competence in using ‘open discovery questions’ (a key healthy conversation skill), participants were provided with four written statements made by hypothetical clients about difficulties with changing health behaviours. Participants were asked to provide individual written responses to these statements mimicking what their verbal response would be if a client made this comment to them whilst they were providing care. Responses to assess competence were double-coded by two researchers (WTL and JLH or LK) using an existing HCS coding matrix to ensure consistency of coding (see Additional file 3). Coding discrepancies were reviewed by the two researchers and discussed until an agreement was reached. The measures of perceived confidence, importance and usefulness of having behaviour change conversations consisted of five questions with responses reported on a 10-point Likert scale, where one was the lowest score (not confident) and ten was the highest (very confident) (see Additional file 4). The measure of importance was included to gauge the priority health professionals give to behaviour change conversations with clients as one of many competing priorities, and to determine their level of receptivity to training in skills that support behaviour change. These evaluation tools and coding matrix have been previously used and reported to evaluate HCS [10, 11, 15, 31].

The feasibility, acceptability and appropriateness of incorporating HCS within professional roles were measured in the follow-up survey using valid and reliable measures of these implementation outcomes [23]. The survey included 12 items (four for each construct) assessed on a 5-point Likert scale (completely disagree (1) to completely agree (5)) (see Additional file 5). Participants’ feedback on the quality of the training was assessed in the post-training survey. Questions to gather their views on using HCS in their role and on implementation strategies to support HCS use in routine care were included in the post-training and follow-up surveys.

In the follow-up telephone interview, participants were asked to reflect on their use of HCS and share experiences of behaviour change conversations they had since the training. The interviewer followed a semi-structured script (see Additional file 6) prompting the participant to describe how a healthy conversation started; what open discovery questions they used (Skill 1); who did most of the talking/listening in the conversation (Skill 3); how they helped the person plan for change by supporting them to set Specific, Measurable, Action-oriented, Realistic, Timed, Evaluated, Reviewed (SMARTER) goals (Skill 4); how they felt the conversation went, and what worked well. The participants were then asked to reflect more broadly on how their practice had changed, how they had used the skills learnt on the course and what they might now do differently (Skill 2). Verbal informed consent to participate in the telephone interview and for audio-recording was sought at the beginning of the interview. The recordings were double-coded by two researchers using an established competency rating rubric to assess demonstrated level of competence in each of the HCS competencies (Fig. 1).

### Analysis

Participants were categorised as working in a clinical setting (e.g. clinical midwife consultant/educator, registered midwife, Aboriginal health worker, allied health practitioner, nurse practitioner, and medical doctor), or non-clinical setting (e.g. population health staff, university lecturer, researcher, and undergraduate/postgraduate university student). Those holding partial clinical roles (with a partial non-clinical role) were categorised to the clinical setting group, as they would have opportunities to practice HCS during client consultations.

Median values were calculated for each TDF domain by summing the scores for each item within the domain and dividing by the total number of items. TDF items that were worded negatively were inverted before being added to the composite totals. Median scores for each TDF domain were calculated and used to categorise domains as potential barriers or enablers; a lower score suggested that the particular domain may be a barrier (< 6) and a higher score (≥6) suggested a perceived enabler to having behaviour change conversations [32]. Reduced TDF responses were obtained post-training due to a coding error in survey data collection (15 participants were not asked these questions). Relationships between domains were explored using pairwise correlation coefficients. Strength of association is described as very high (0.90-1.00), high (0.70-0.90), moderate (0.50-0.70), low (0.30-0.50 and negligible (0.00-0.3), with corresponding negative correlations [33]. Only moderate and higher correlations are reported.

Descriptive analyses were used to report demographic values. Differences between scores at the various time points for the TDF barriers were analysed by Wilcoxon matched-pairs signed-rank test to account for the smaller sample size and compared pre-training to both post-training and follow-up scores. To ensure accuracy of interpretation and account for missing data, a second analysis using first observation carried forward was also performed. Changes in confidence are displayed graphically, by constructing 25-75th centile values and using median values over the three time points.

## Results

### Participant characteristics

Sixty-four participants consented to complete the pre-training survey and 62 completed the post-training survey (Table 1). Thirty-four participants completed the follow-up survey, and six completed a telephone interview. Due to the low uptake of the telephone interview, a decision was made not to analyse these data.

**Table 1:** Characteristics of participants (*n* = 64) presented by clinical and non-clinical role

	Working in a clinical setting^**a**^		Working in a non-clinical setting		Total	
	n	(%)	n	(%)	n	(%)
**Participant survey completion**						
Pre-training survey	37	(57.8%)	27	(42.2%)	64	(100%)
Post-training survey	36	(58%)	26	(42%)	62	(100%)
Follow-up survey	16	(48%)	17	(52%)	34	(100%)
**Training location**						
Newcastle	24	(37.5%)	23	(31.3%)	47	(73.4%)
Tamworth	13	(20.3%)	4	(6.3%)	17	(26.6%)
**Identify as Aboriginal and/or Torres Strait Islander**						
Aboriginal origin	7	(10.9%)	3	(4.7%)	10	(15.6%)
Neither	29	(45.3%)	24	(37.5%)	53	(82.8%)
Do not want to answer	1	(1.6%)	0	(0.0%)	1	(1.6%)
**Sex**						
Women	36	(56.3%)	26	(40.6%)	62	(96.9%)
Men	1	(1.6%)	1	(1.6%)	2	(3.1%)
**Years of experience in current professional position**						
≤ 2 years	9	(14.1%)	6	(9.4%)	15	(23.4%)
3-4 years	9	(14.1%)	6	(9.4%)	15	(23.4%)
5-9 years	9	(14.1%)	3	(4.7%)	12	(18.8%)
≥ 10 years	10	(15.6%)	12	(18.8%)	22	(34.4%)
**Conducts research in current professional position**						
Yes	10	(15.6%)	23	(35.9%)	33	(51.6%)
**Reason/s for attending training (multiple responses could be selected)**						
To improve communication skills with clients	31	(48.4%)	13	(20.3%)	44	(68.8%)
To incorporate HCS in my teaching	7	(10.9%)	9	(14.1%)	16	(25.0%)
To incorporate HCS in my research	4	(6.3%)	16	(25.0%)	20	(31.3%)
Other	7	(10.9%)	2	(3.1%)	9	(14.1%)

^a^Participants were categorised as ‘working in a clinical setting’ (e.g. clinical midwife consultant/educator, registered midwife, Aboriginal Health Worker, allied health practitioner, nurse practitioner, and medical doctor), or working in a non-clinical setting (e.g. Population Health staff, university lecturer, researcher, undergraduate/postgraduate university student). Those holding partial clinical roles (and a partial non-clinical role) were also categorised to the clinical setting group, as they would have opportunities to practise HCS during client consultations

Most participants were female (97%), and 16% identified as Aboriginal or Torres Strait Islander origin. Participants were primarily health professionals employed by NSW public health services (*n* = 39) and/or University academics (teaching and/or research staff) (*n* = 23; data not shown in table). Others were employed by a medical research institute, non-government organisation or in private practice (*n* = 9). One third of participants had at least 10 years’ experience in their health profession. Participants could select multiple reasons for attending HCS training, with the most commonly reported being to: i) improve communication skills with clients (69%), ii) include HCS in teaching of undergraduate health professional students (31%), or iii) use HCS in their research (25%).

### Changes in scores for the TDF barriers and enablers

Pre-training, six of the eight domains were identified as potential barriers to having behaviour change conversation (median domain score < 6.0) (Table 2): these were skills; beliefs about capabilities; intentions; goals; memory, attention and decision processes; and behavioural regulation. The domains of social/professional role and identity, and beliefs about consequences had high scores pre-training (median domain score ≥ 6.0), suggesting that they may be enablers to having behaviour change conversations. Seven of the eight domains scores significantly improved post-training (meaning they were less likely to be identified as a barrier post-training), with skills, belief about capabilities and goals domains remaining significantly higher at 6-10 weeks post-training follow-up. Findings were consistent with the secondary analysis using first observation carried forward for missing data (not reported).

**Table 2:** Participants’ median scores for the theoretical domains framework barriers at pre-training, post-training and follow-up

Domain	Definition	Number of items	Pre-training (***n*** = 63)	Post-training (***n =*** 47) ^1^	Follow-up (***n*** = 33)	Sign rank	Sign rank
			Median (IQR)	Median (IQR)	Median (IQR)	Pre-, post	Pre-, follow up
Skills	An ability or proficiency acquired through practice	3	4.7 (3.3-5.3)	6.0 (5.7-6.7)	5.7 (5.3-6.0)	*p <* 0.01*	*p <* 0.01*
Social/professional role and identity	A coherent set of behaviours and displayed personal qualities of an individual in a social or work setting	4	6.0 (5.5-7.0)	6.5 (6.0-7.0)	6.0 (5.5-7.0)	*p <* 0.01*	*p* = 0.38
Beliefs about capabilities	Acceptance of the truth, reality, or validity about an ability, talent, or facility that a person can put to constructive use	3	4.7 (3.3-6.0)	6.0 (5.3-6.3)	5.7 (5.0-6.0)	*p <* 0.01*	*p <* 0.01*
Beliefs about consequences	Acceptance of the truth, reality, or validity about outcomes of a behaviour in a given situation	2	6.0 (5.0-6.5)	6.5 (6.0-7.0)	6.0 (5.0-7.0)	*p <* 0.01*	*p* = 0.14
Intentions	A conscious decision to perform a behaviour or a resolve to act in a certain way	4	5.4 (4.4-6.4)	6.5 (5.9-7.0)	5.8 (5.1-6.3)	*p <* 0.01*	*p* = 0.23
Goals^2^	Mental representations of outcomes or end states that an individual wants to achieve	3	4.3 (3.7-5.0)	5.3 (5.0-5.7)	4.7 (4.3-5.3)	*p <* 0.01*	*p* = 0.01*
Memory, attention and decision processes	The ability to retain information, focus selectively on aspects of the environment and choose between two or more alternatives	4	4.3 (4.0-5.0)	4.8 (4.0-5.3)	4.8 (4.3-5.3)	*p* = 0.16	*p* = 0.24
Behavioural Regulation^2^	Anything aimed at managing or changing objectively observed or measured actions	3	4.0 (3.0-4.7)	4.7 (4.0-5.3)	4.7 (3.7-5.0)	*p <* 0.01*	*p* = 0.05

^1^*n =* 47 at post-training survey due to incomplete survey responses; ^2^ discriminant content validity of the items measuring these domains was not demonstrated in Huijg et al. [26]. *A score of 1 = strongly disagree/not strong at all/never, and a score of 7 = strongly agree/very strong/always*

Eight moderate correlations between TDF domains scores were seen in pre-training TDF domains (see Additional file 7). Of note at pre-training was the moderate correlation between skills and belief about capabilities (0.68, *p* < 0.05). At post-training, seven moderate correlations between TDF domains were identified, which were not always consistent with the pre-training associations, with the strongest correlation existing between beliefs about consequences and social/professional role and identity (0.65, *p <* 0.05).

### Changes in competence

**Fig. 2:**
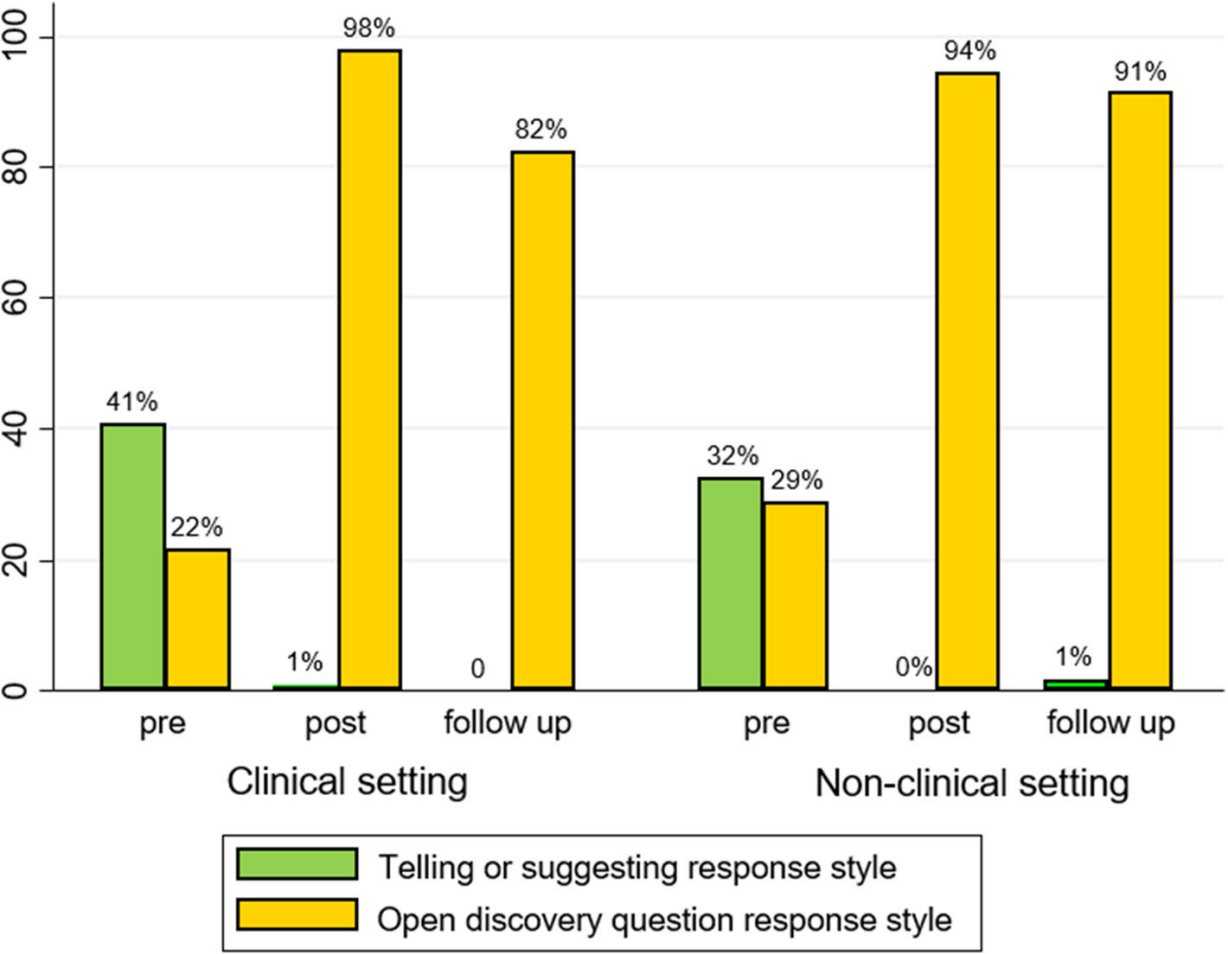
The impact of HCS training on participant response style practices

Figure 2 outlines the impact of HCS training on participants’ responses to four written statements that clients could make about difficulties with changing behaviour, according to those working in clinical or non-clinical settings. The desired outcome of the training is to see a shift from telling/suggesting to asking open discovery questions, indicating a more empowering approach. Prior to training 37% of responses were telling/suggesting, 12% reflection/empathy, 18% closed questions, and only 25% open discovery questions (see Additional file 8). The proportion of responses that were open discovery questions increased from pre- to post-training (96%, *p* < 0.001), and remained significantly higher than pre-training at follow-up (87%, *p <* 0.001).

*Types of response provided by participants to four written statements about behaviour change immediately before (*n* = 64) and after training (*n* = 62), and at 6-10 weeks follow-up (*n* = 34). See Additional file 8 for a full list of types of responses at pre, post and follow-up time points.

### Changes in confidence, importance and usefulness

**Fig. 3:**
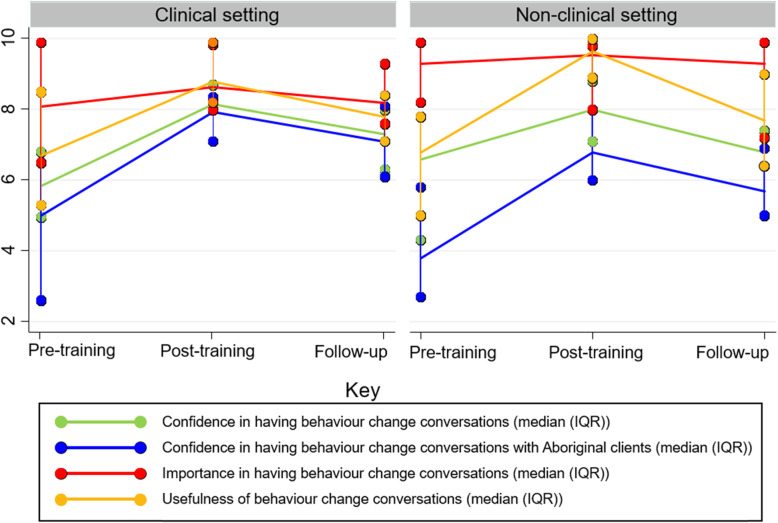
Participants’ confidence, importance and usefulness of having behaviour change conversations, by clinical and non-clinical role

Changes in participants’ self-reported confidence, importance and value of having behaviour change conversations are presented in Fig. 3, according to those working in clinical or non-clinical settings. Regardless of setting, participants consistently rated the importance of supporting clients to change their behaviour as very high (median score of ≥8/10) at all time points. Both groups reported greater usefulness of having behaviour change conversations post-training, which reduced but remained higher than pre-training levels at follow-up. At follow-up, participants felt that HCS were useful for supporting Aboriginal clients to make behaviour changes (median (IQR) = 7.1 (5.9-8.4) out of 10) (data not shown in figure). Participants also reported increased confidence in supporting clients to make behaviour changes post-training, including an increased confidence in having behaviour change conversations with Aboriginal clients. Those working in clinical settings maintained higher confidence in supporting clients to make behaviour changes at follow-up than those in non-clinical settings.

### Feasibility, acceptability and appropriateness measures

Results presented in Table 3 are composite scores of statements that participants used to rate the acceptability, appropriateness and feasibility of using HCS in their professional role at follow-up. HCS was rated as very acceptable (median: 4.75/5), which included ratings of appeal, approval, likeability, and welcoming the opportunity to use HCS in behaviour change conversations with clients. Participants indicated that use of HCS was appropriate (median: 5/5), meaning the skills were fit for purpose, suitable, applicable and overall a good match for their needs in their roles. They also indicated that HCS were feasible (median: 4.25/5), suggesting that HCS are implementable, possible, doable and easy to use in their positions.

**Table 3:** The acceptability, appropriateness and feasibility of using HCS in practice (score out of five)

Construct	Median	IQR
Acceptability of intervention measure score	4.75	4-5
Intervention appropriateness measure score	5	4-5
Feasibility of intervention measure score	4.25	4-5

### Training satisfaction and feedback

Participants (*n* = 61) rated their satisfaction with the training as very high (median (IQR) = 4.9/5 (4.4-5.0)). They reported HCS to be a sustainable communication style that could be used in the workplace (6.6/7 (5.8-6.7)). Participants felt that they could apply HCS to various types of behaviour change conversations within their role, including but not limited to: healthy eating (*n* = 55; 89%); maintaining a healthy weight (*n* = 52; 84%); physical activity (*n* = 50; 81%); alcohol consumption (*n* = 47; 76%); mental and emotional health (*n* = 46; 74%); tobacco use (*n* = 43; 69%); drug use (*n* = 31; 58%); and breastfeeding (*n* = 36; 58%). Other areas to which participants reported (via open text) that they could apply HCS were domestic violence, medication management, oral health, developing a birth plan, and to support student health professional development and supervision. The top three preferences to further support them to use HCS were: i) online refresher training (53%); ii) having a HCS buddy to reflect with (53%) and iii) additional resources (50%). One third of participants indicated a preference for face-to-face refresher training, however participants had limited interest in: i) including HCS in their annual performance review (6%); follow-up telephone meetings with a trainer (9%); or connecting with participants via a social media group (18%). When asked about applicability of HCS training for their colleagues, 92% of participants felt that ‘all’ or ‘most’ of their colleagues would benefit from the training.

## Discussion

This is the first study to apply an implementation determinants framework to investigate the impact of HCS training on health professionals’ barriers to having behaviour change conversations in clinical and non-clinical settings. HCS training addressed the barriers defined by the TDF domains of skills, belief about capabilities, and having a goal (i.e. outcome that they want to achieve) for how to have behaviour change conversations in practice 6-10 weeks post-training. Changes in beliefs about capability are consistent with evidence published to date on HCS training in relation to increased confidence, as both can be seen as measures of self-efficacy [10, 15]. It was anticipated that the TDF domains of skills and goals would improve, given that the training consists of multiple opportunities to practise the skills and set goals for future professional practice. Additional file 1 demonstrates how a range of behaviour change techniques underpin the training and are intended to facilitate changes including those seen in the TDF domains. More broadly, current findings improve our understanding on how behaviour change communication training aligned with the theoretical determinants of their own behaviour, and can increase health professionals likelihood to engage in behaviour change conversations with their clients [34].

Application of the TDF identified the domains of skills; beliefs about capabilities; intentions; goals; memory, attention and decision processes; and behavioural regulation to be pre-training barriers to having behaviour change conversations with clients. This is consistent with previous research [2, 18–20] suggesting that training targeting cross-disciplinary barriers and enablers could be an efficient capacity-building strategy to support health professionals from different disciplines to use person-centred approaches [2]. Our findings that the domains of social/professional role and identity, and beliefs about consequences are enablers align with some previous findings [2, 34] that health professionals in a variety of clinical and non-clinical roles perceive it as their role to support clients to make behaviour changes, and believe that having these conversations could improve their clients’ health.

Intentions (related to a conscious decision to perform a behaviour) to have behaviour change conversations improved immediately post-training, but this was not sustained at 6-10 week follow-up. This could indicate a need for an additional activity in the HCS training or for ongoing support based on implementation intentions [35]. This would prompt trainees to make an “if-then plan”, which would be expected to maintain their intention to use HCS in their future practice. Improvements in behavioural regulation similarly were not sustained at follow-up, and the memory, attention and decision processes domain did not change at any time point. This might have occurred because these domains relate to longer-term behaviour determinants such as memory, attention, automaticity, self-monitoring and action planning which may require additional support post-training. The majority of trainees indicated that this would best be provided by way of online refreshers and buddy networks. These domains may also be connected to organisational culture, which may require changes to clinical protocols and procedures to support health professionals to embed HCS in practice. To assess the effectiveness of these strategies it would then be important to measure skill use over a longer period of time than was assessed in this study.

Consistent with previous HCS evaluations [10, 14, 15], the training was highly successful in changing participants’ short and medium term competence in using open discovery questions, indicating an ability to adopt a more exploratory approach to understand the context of a person’s behaviour, and to support them to plan their own solutions. In HCS training, participants are supported to explore the potential outcomes of using other response styles: telling/advice-giving can feel overwhelming or patronising, and fails to find out anything about the individual; sharing the experiences of the practitioner or others can demonstrate understanding, but can also be seen as collusion and not lead to change; expressing empathy can help build rapport, but again is not supporting the identification of strategies for change; asking closed questions risks shutting the conversation down; asking ‘why’ can make people feel judged and defensive. Therefore, facilitating participants to recognise the value of using open discovery questions (beginning with ‘what’ and ‘how’) to explore someone’s world and support identification of first steps to change is one of the most important outcomes of HCS training. Higher confidence levels were maintained by health professionals working in clinical settings compared to non-clinical settings. This may be because these participants had more opportunities to regularly use HCS in their patient-facing roles. Health professionals reported lower pre-training confidence in having behaviour change conversations with Aboriginal clients, which improved and was sustained post-training. The Aboriginal cultural review process identified that HCS philosophy and training align with how Aboriginal people engage and understand each other’s world. HCS has the potential to improve engagement of non-Aboriginal health professionals with Aboriginal people by exploring the social determinants of health, and building on strengths to facilitate empowerment and autonomy in finding solutions. The consistency of the findings with previous HCS evaluations [10, 14, 15] demonstrates the robustness of HCS training in eliciting improved competence and confidence outcomes in participants from a range of health professions, working in a variety of settings across numerous countries, while being trained by different facilitators taught through a train-the-trainer model.

Promisingly, the results show that HCS training was very well received. Participants reported HCS to be a highly acceptable communication style, appropriate for supporting clients to make behaviour changes, and feasible to use within their role. They recognised that HCS can be applied to a wide variety of behaviour change topics, demonstrating the broad benefit of HCS both to support clients with all forms of health behaviour change, and students’ health professional development and supervision. These findings provide further valuable evidence of the effectiveness and suitability of HCS training in different contexts and with different health professional populations. Future research could examine changes in barriers and enablers to behaviour change conversations in other health professional groups, including undergraduate health professional students who may experience different barriers and enablers to having behaviour change conversations. Studies assessing implementation processes and outcomes (such as adoption, acceptability, appropriateness, feasibility, reach, fidelity, cost, and sustainment) could assist the implementation of HCS in other clinical, health service and teaching research projects more broadly by advancing our understanding of the process of implementing HCS at scale, and compare the effectiveness of different implementation strategies (e.g. face-to-face versus online training) to enhance the efficiency of disseminating HCS [36]. Since behaviour change conversations are key approaches to health promotion in clinical practice guidelines, and many health professionals report barriers to engaging in these conversations with clients, future research should investigate if HCS training improves health professionals’ adherence to recommended care guidelines, and its impact on client health behaviours and clinical outcomes.

The study had a number of limitations. A convenience sampling approach was used to recruit participants to undertake the training. This approach was used due to their interest, and experience in the clinical setting and/or educator roles as many participants also completed the HCS train-the-trainer program to learn how to train other staff, colleagues and students in HCS. Current findings may not therefore be generalisable to the experiences of other health professionals in the general health service, teaching or research workforce. However, there is no reason to believe that the views expressed by those taking part are likely to be very different if another group of health professionals had been recruited. It was not possible to include a control group within the study design, thus we are unable to draw definitive conclusions that changes in barriers to having behaviour change conversations are due to training completion. Future evaluations of HCS training should endeavour to use a more controlled study design. Response rates were high for pre- and post-training surveys, however the response rates for the follow-up survey (53%) and telephone interview (9%) were low; influencing the decision not to report the telephone interview data here. The latter may reflect findings that only 9% of participants wanted follow-up telephone meetings with a trainer, therefore alternative methods should be considered in the future, both for evaluation purposes and to support skill development. Participants may have felt some responsibility to provide socially desirable survey responses, however this risk was mitigated by participants completing all surveys independently and anonymously. The TDF was not used to prospectively design HCS training to address barriers and enablers to having behaviour change conversations. Rather we identified key barriers to having behaviour change conversations through the literature, and surveys and consultations with local clinical services, and mapped these to eight TDF domain to limit survey length and participant burden. It is possible that the five TDF domains not included in the survey could be barriers or enablers to health professionals having behaviour change conversations, which may or may not have been impacted by the training. Previous research has shown the TDF survey to be a useful and valid tool for assessing changes in barriers and enablers, and is able to discriminately assess the majority of TDF domains, with the exception of goals and behavioural regulation [26].

This is the first evaluation of HCS in an Australian context, and the first to map HCS training to the TDF to understand the mechanism/s of action. This will enable further refinement of the training to enhance HCS use and thus embed person-centred behaviour change conversations in routine practice. A further strength of the current study was the use of the TDF [24], and validated surveys to assess barriers and enablers [26] and acceptability, appropriateness and feasibility of HCS [23]. Using measures of competence, confidence, importance and usefulness previously developed and published by the original HCS training team enables comparison of findings with previous evaluations of HCS, demonstrating the robustness of HCS training in producing desired outcomes. All aspects of the project underwent cultural review to ensure cultural safety and appropriateness for Aboriginal health professionals and people.

## Conclusion

Health professionals’ competence in using HCS and confidence in having behaviour change conversations increased post-training. HCS training increased health professionals’ self-reported skill level and belief in their capability to have behaviour change conversations, and their goals about having a behaviour change conversation in practice, up to 6-10 weeks post-training. HCS training activities and post-training implementation strategies could be refined to maintain health professionals’ behavioural regulation and intentions to use HCS, and to remind them to use HCS when supporting their clients to make a change. Findings from the current study suggest that broad implementation of HCS training may be an effective capacity-building strategy to support health professionals to use a person-centred, empowering approach to behaviour change, including with Aboriginal people, to improve population health.

## Supplementary Information

**Additional file 1:.** Healthy Conversation Skills training activities mapped to the Taxonomy of Behaviour Change Techniques.

**Additional file 2:.** Theoretical Domains Framework Survey Questions.

**Additional file 3:.** Coding matrix for responses to quotes for Healthy Conversation Skills Training.

**Additional file 4:.** Healthy Conversation Skill confidence, importance, usefulness and competence questions.

**Additional file 5:.** Acceptability, appropriateness and feasibility questions.

**Additional file 6:.** Healthy Conversation Skills follow-up telephone intervention protocol.

**Additional file 7:.** Moderate, high and very high correlations of the pre and post Theoretical Domains Framework domains in participants from the Healthy Conversation Skills training.

**Additional file 8:.** The impact of HCS training on practice as reported by the types of response provided by participants.

## Data Availability

The datasets used and analysed during this study are available from the corresponding author on reasonable request.

## Abbreviations

HCS: Healthy Conversation Skills
IQR: Interquartile Range
RCT: Randomised Controlled Trial
TDF: Theoretical Domains Framework
