# Supplementary material for: The impact of Healthy Conversation Skills training on health professionals’ barriers to having behaviour change conversations: a pre-post survey using the Theoretical Domains Framework
Source: BMC Health Serv Res. 2021 Aug 27;21:880. doi: 10.1186/s12913-021-06893-4 (PMC8394191; doi:10.1186/s12913-021-06893-4)
Supplement: Supplementary file 1 — Additional file 1:. Healthy Conversation Skills training activities mapped to the Taxonomy of Behaviour Change Techniques. [file 12913_2021_6893_MOESM1_ESM.docx]

**Additional file 1.** Healthy Conversation Skills training activities mapped to the Taxonomy of Behaviour Change Techniques.

| **Activity** | **Group No.** | **BCT Group** | **BCT No.** | | **BCT** | **Activity component** |
| --- | --- | --- | --- | --- | --- | --- |
| Overall, throughout both sessions | 1 | Goals & planning | 1.2  1.6 | | Problem-solving  Discrepancy between current behaviour & goal | How to use skills in practice – prompting trainee to generate/select strategies to overcome barriers & increase facilitators to HCS use, including “relapse prevention” & “coping planning” to avoid returning to any previous style of interacting.  Reflection on changing current practice – drawing trainee’s attention to discrepancies between current practice and plans/goals to incorporate HCS into practice. |
|  | 3 | Social support | 3.2  3.3 | | Social support (practical)  Social support (emotional) | Group training & pair work provides practical support (listening & sharing tips) for practising HCS in the training & later in the workplace.  Group training & pair work provides emotional support (encouragement/praise) for practising HCS. |
|  | 4 | Shaping knowledge | 4.1  4.2 | | Instruction on how to perform the behaviour  Information on antecedents | Skills training, including advice & agreement for how to develop questions, support SMARTER planning etc.  Review what predicts behaviour, eg when running out of time can revert to telling or suggesting. |
|  | 6 | Comparison of behaviour | 6.1 | | Demonstration of the behaviour | HCS modelled by facilitator in all activities, & by trainees in various real/role play activities. |
|  | 7 | Associations | 7.1 | | Prompts & cues | Provided in the training room & as hand-outs. Also attention drawn to language used by facilitator, ie modelling HCS. |
|  | 8 | Repetition & substitution | 8.1  8.3  8.6 | | Behavioural practice/rehearsal  Habit formation  Generalisation of a target behaviour | Prompt practice of all HCS in training room (artificial but safe environment).  Prompt practice of all HCS in real world (more challenging but real).  If have tried using skills with friend/relative, encourage to try out skills in workplace (eg with colleagues, patients/clients). |
| A1 Wider Determinants of Health & Exploratory Discussion | 6 | Comparison of behaviour | 6.2 | | Social comparison | 1^st^ opportunity to compare own practice & experiences with others. |
|  | 13 | Identity | 13.3 | | Incompatible beliefs | Draws attention to discrepancies between current/ past behaviour and view of self as health professional for example. So when have challenges & frustrations around supporting someone, may lead to self-doubt regarding beliefs about competence. |
| A2.1 & 2.2 Recording & Listening back | 1 | Goals & planning | 1.2 | | Problem-solving | Listening to recordings elicits reflection on current practice, areas for improvement & strategies to develop skills. |
|  | 2 | Feedback & monitoring | 2.2  2.7 | | Feedback on behaviour  Feedback on outcome(s) of behaviour | Listening back provides opportunity for partner to provide evaluative feedback on performance.  Listening back is opportunity to discuss & receive feedback on what happened as a result of asking any ODQs as opposed to telling/suggesting or asking closed Qs. |
|  | 3 | Social support | 3.2  3.3 | | Social support (practical)  Social support (emotional) | Working in pairs to undertake & review recording task, elicits:  tips for practice & & sharing task (3.2);  someone listening & offering praise & encouragement (3.3). |
|  | 6 | Comparison of behaviour | 6.2 | | Social comparison | Listening back to self & partner allows comparison of both performances & outcomes related to these. |
|  | 13 | Identity | 13.2  13.3 | | Framing/reframing  Incompatible beliefs | Listening back following trainer demonstration of skills provides a new perspective on using skills hence changing cognitions related to using the skills.  Draws attention to discrepancies between current/ past behaviour and self-image as competent practitioner, in order to create discomfort. |
| A3 Beliefs Axis | 1 | Goals & planning | 1.2 | | Problem-solving | Encouraging analysis of factors influencing past/current behaviour in relation to beliefs, leads to shifts in understanding and intentions to change behaviour. |
|  | 13 | Identity | 13.2  13.3 | | Framing/reframing  Incompatible beliefs | Exploring own beliefs & listening to counter-arguments provides new perspectives about current practice in order to change cognitions & emotions about changes to behaviour & skills required.  Draws attention to discrepancies between beliefs and past behaviour, in order to create discomfort & elicit new thinking. |
| A4 Response Styles | 1 | Goals & planning | 1.2 | Problem-solving | | Allows trainees to analyse own responses and factors influencing these, leading to generation of strategies to use new skills. |
|  | 4 | Shaping knowledge | 4.1 | Instruction on how to perform the behaviour | | Skills training supporting trainee to explore/agree on how to perform the behaviour, ie using ODQs. |
|  | 8 | Repetition & substitution | 8.1  8.2  8.4 | Behavioural practice/rehearsal  Behaviour substitution  Habit reversal | | Prompting use of ODQs in the training context in order to increase habit & skill.  Prompting substitution of the unwanted behaviour (suggesting/telling etc) with ODQs.  Prompt rehearsal of ODQs to replace habitual use of suggestions/information-giving etc. |
|  | 9 | Comparison of outcomes | 9.3 | Comparative imagining of future outcomes | | Prompt comparison of possible outcomes following either suggesting/telling or asking ODQs, ie where does the conversation go? which is more effective for changing behaviour? |
|  | 13 | Identity | 13.2  13.3 | Framing/reframing  Incompatible beliefs | | Encouraging adoption of new perspective on using ODQs rather than other responses, in order to change cognitions about doing this – where it takes the conversation.  Responding to quotes highlights discrepancies between current behaviour (telling/suggesting etc) and self-image as someone who explores someone’s world & wants to support change. |
| A5 Setting SMARTER Goals | 1 | Goals & planning | 1.1  1.2 | Goal-setting (behaviour)  Problem-solving | | Working through example of setting/planning a behavioural goal.  Prompting exploration & analysis of factors influencing the behaviour & strategies to overcome barriers & facilitate action. |
|  | 8 | Repetition & substitution | 8.1 | Behavioural practice/rehearsal | | Prompting use of HCS, including ODQs & SMARTER goal-setting within training context in order to increase habit & skill. |
|  | 13 | Identity | 13.2 | Framing/reframing | | Prompting adoption of a new perspective regarding making goals “SMARTER” & “owned” by individual in order to change cognitions about effectively supporting goal-setting. |
| A6 SMARTER Planning for Change | 1 | Goals & planning | 1.1  1.2  1.4  1.8  1.9 | Goal-setting (behaviour)  Problem-solving  Action-planning  Behavioural contract  Commitment | | Setting & agreeing behavioural goals in pairs.  In pairs, prompting each other to identify and analyse factors influencing behaviour & generating strategies to overcome barriers & facilitate change.  In pairs, prompting detailed (SMARTER) planning of behaviour.  Individual creates a written specification of the behaviour to be performed by completing the SMARTER planning for change sheet.  By asking individuals to state their goals, or their intention to reach their goal, it elicits a commitment to change their behaviour. |
|  | 2 | Feedback & monitoring | 2.2  2.3 | Feedback on behaviour  Self-monitoring | | By working in pairs, individuals can provide evaluative feedback to each other on performance of the behaviour (setting a SMARTER goal)  As part of SMARTER planning individuals can be supported to identify ways of monitoring changes made. |
|  | 3 | Social support | 3.2  3.3 | Social support (practical)  Social support (emotional) | | Working in pairs to support each other to set a SMARTER goal may include:  questions to encourage reflection on barriers/ solutions etc.  praise, encouragement, listening, empathy & sharing experiences. |
|  | 7 | Associations | 7.1 | Prompts/cues | | SMARTER planning for change sheet provides prompts/cues for setting goal(s). |
|  | 8 | Repetition & substitution | 8.1 | Behavioural practice/rehearsal | | Working in pairs provides each with the opportunity to practise asking ODQs, listening, supporting SMARTER planning & reflecting on usefulness of HCS. |
|  | 15 | Self-belief | 15.1  15.2  15.3  15.4 | Verbal persuasion about capability  Mental rehearsal of successful performance  Focus on past success  Self-talk | | Working in pairs provides opportunity for using a range of strategies to boost self-belief, inc challenging self-doubts (15.1), imagining successful performance of behaviour (15.2), reviewing past successes (15.3), prompting positive self-talk before & during behaviour (15.4). |
|  | 16 | Covert learning | 16.2 | Imaginary reward | | Working in pairs provides opportunity to imagine performing the behaviour in real-life followed by a pleasant/desirable consequence (eg empowerment of patient following a HC; increased job satisfaction; healthier patient). |
| A7 Reviewing SMARTER Planning for Change sheets & Practising HCS | 1 | Goals & planning | 1.2  1.5  1.6  1.9 | Problem-solving  Review behaviour goal(s)  Discrepancy between current behaviour & goal  Commitment | | In pairs, prompting each other to identify and analyse factors influencing behaviour & generating strategies to overcome any barriers & facilitate further change.  In pairs jointly review goal(s) & consider modifications to strategies or goal(s).  In pairs jointly review discrepancies between current behaviour (inc context, frequency, duration, intensity) & previously set goal(s) or action plans.  In pairs, can support each other to reaffirm commitment to continue/restart change. |
|  | 2 | Feedback & monitoring | 2.2  2.3 | Feedback on behaviour  Self-monitoring of behaviour | | In pairs jointly engage in reflective feedback on performance of the behaviour.  SMARTER planning for change sheet is one method of recording behaviour change strategies & plans |
|  | 3 | Social support | 3.2  3.3 | Social support (practical)  Social support (emotional) | | Working in pairs to support each other to review a SMARTER goal may include:  questions to encourage reflection on barriers/ solutions etc.  praise, encouragement, listening, empathy & sharing experiences. |
|  | 7 | Associations | 7.1 | Prompts/cues | | SMARTER planning for change sheet provides prompts/cues for reviewing/revising goal(s). |
|  | 8 | Repetition & substitution | 8.1  8.3  8.6  8.7 | Behavioural practice/rehearsal  Habit formation  Generalisation of target behaviour  Graded tasks | | Working in pairs provides each with the opportunity to practise asking ODQs, listening, supporting SMARTER planning & review of goal(s), reflecting on usefulness of HCS.  Working in pairs provides opportunity to prompt rehearsal of behaviour in the same context so the context elicits the behaviour (eg using HCS with every patient).  Working in pairs provides opportunity to prompt performance of wanted behaviour in different situations (eg used HCS with own family, now start to incorporate into professional practice).  In pairs can review how to set increasingly difficult, but realistic tasks, to work towards performance of the behaviour & achievement of goal(s). |
|  | 13 | Identity | 13.3 | Incompatible beliefs | | In pairs can review an discrepancies between current behaviour & self-image in order to create discomfort (eg if not undertaken desired change, but sees themselves as a person who does the desired behaviour). |
|  | 15 | Self-belief | 15.1  15.2  15.3  15.4 | Verbal persuasion about capability  Mental rehearsal of successful performance  Focus on past success  Self-talk | | Working in pairs provides opportunity for using any of these strategies to boost self-belief, eg challenging self-doubts (15.1), imagining successful performance of behaviour (15.2), reviewing past successes (15.3), prompting positive self-talk before & during behaviour (15.4). |
|  | 16 | Covert learning | 16.2 | Imaginary reward | | Working in pairs provides opportunity to imagine performing the behaviour in real-life followed by a pleasant/desirable consequence (eg empowerment of patient following a HC; increased job satisfaction; healthier patient). |
| A8 Introduction to BCTs | 3 | Social support | 3.2  3.3 | Social support (practical)  Social support (emotional) | | Working in small groups to support each other to review use of BCTs may include:  Asking questions to encourage reflection on barriers to use / past experiences etc (3.2);  listening, empathy & sharing experiences (3.3). |
|  | 9 | Comparison of outcomes | 9.1  9.3 | Credible source  Comparative imagining of future outcomes | | Provide list of BCTs identified in the literature as being generally effective in supporting change.  Prompt trainees to imagine & compare possible outcomes of using HCS to support patients to incorporate BCTs into change plans, ie more likely to make sustainable changes. |
|  | 15 | Self-belief | 15.3 | Focus on past success | | Working in small groups provides opportunity to reflect on previous successes in using BCTs to support behaviour change. |
| A9 SMARTER Team Challenge | 1 | Goals & planning | 1.1  1.2  1.4 | Goal-setting (behaviour)  Problem-solving  Action-planning | | Opportunity to practise asking ODQs to support SMARTER goal-setting.  Opportunity for everyone to analyse factors influencing behaviour and generate questions to support behaviour change.  Opportunity to prompt detailed planning of performance of the behaviour. |
|  | 2 | Feedback & monitoring | 2.2 | Feedback on behaviour | | Each team provides evaluative feedback on the performance of the other team, ie how successfully they supported SMARTER goal-setting. |
|  | 3 | Social support | 3.3 | Social support (emotional) | | Taking a team approach to supporting SMARTER goal-setting provides a safe, shared environment to practise HCS. |
|  | 8 | Repetition & substitution | 8.1 | Behavioural practice/rehearsal | | Opportunity to practise all 4 HCS. |
|  | 10 | Reward & threat | 10.4 | Social reward | | Facilitator (& other team) can congratulate both teams on effort & progress in performing required behaviours – using HCS to support SMARTER goal-setting. |
| A10 Creating own Resource – combining HCS, philosophy & BCTs | 1 | Goals & planning | 1.2 | Problem-solving | | In small groups, reflecting on HCS training overall & how to most effectively graphically represent this to prompt future HCS use. |
|  | 3 | Social support | 3.2  3.3 | Social support (practical)  Social support (emotional) | | Working in small groups to support each other to design a resource includes use of:  questions to encourage reflection on training.  praise, encouragement, listening, empathy & sharing ideas. |
| Comparison of T1&T2 evaluation sheets | 1 | Goals & planning | 1.2  1.6 | Problem-solving  Discrepancy between current behaviour & goal | | By comparing pre- & post-training sheets, trainees are able to analyse factors influencing their responses and reflect on how to increase use of new skills.  Comparison of sheets draws attention to discrepancies between current practice and goals for implementing HCS in the future. |
|  | 2 | Feedback & monitoring | 2.3 | Self-monitoring of behaviour | | Comparing the self-completed sheets provides opportunity for recording own behaviour, and support their behaviour change strategy. |
|  | 8 | Repetition & substitution | 8.1 | Behavioural practice/rehearsal | | Completing & comparing the sheets prompts practice of the behaviour, eg forming ODQs. |
|  | 13 | Identity | 13.3 | Incompatible beliefs | | Comparing the sheets draws attention to discrepancy between past behaviour & self-image in order to create discomfort. |
| Reflection on training & next steps  Provision of laminated hand-outs | 1 | Goals & planning | 1.1  1.2  1.4  1.8 | Goal-setting (behaviour)  Problem-solving  Action-planning  Commitment | | Trainees encouraged to agree goals for practising HCS.  Trainees prompted to review barriers & facilitators to incorporating HCS into practice (can include ‘relapse prevention’).  Trainees can be encouraged to make detailed plans for practising HCS, eg context, frequency.  Trainees asked to verbalise commitment to changing behaviour & using HCS. |
|  | 3 | Social support | 3.2  3.3 | Social support (practical)  Social support (emotional) | | Facilitator provides social support with:  questions to encourage reflection on barriers/ solutions etc.  praise, encouragement, listening, empathy & sharing tips. |
|  | 7 | Associations | 7.1 | Prompts/cues | | Laminated hand-outs act as social stimulus for prompting HCS use. Trainees encouraged to think about & discuss where they might keep/display them. |
|  | 8 | Repetition & substitution | 8.1  8.3  8.7 | Behavioural practice/rehearsal  Habit formation  Graded tasks | | Prompt trainees to practise HCS at every opportunity in order to increase habit & skill.  Prompt trainees to use HCS in their routine professional practice so that it becomes automatic.  Encourage trainees to set easy-to-perform tasks, making these increasingly difficult but achievable over time, eg build up to using HCS in all situations, even the most challenging. |
|  | 12 | Antecedents | 12.5 | Adding objects to the environment | | Displaying laminated hand-outs in the workplace facilitates performance of the behaviour, HCS use. |
|  | 13 | Identity | 13.1  13.2 | Identification of self as role model  Framing/reframing | | Opportunity to encourage trainee to reflect on role within wider team who might not have been trained.  Opportunity to reflect on adopting a new perspective on professional practice, in order to change cognition about role in supporting change & use of HCS. |
|  | 15 | Self-belief | 15.1 | Verbal persuasion | | Facilitator can empower trainee & raise self-efficacy. |

Dr Wendy Lawrence PhD CPsychol AFBPsS

“Healthy Conversation Skills” lead trainer

November 2016

Rationale HCS activities/BCT table
