# Supplementary material for: The impact of Healthy Conversation Skills training on health professionals’ barriers to having behaviour change conversations: a pre-post survey using the Theoretical Domains Framework
Source: BMC Health Serv Res. 2021 Aug 27;21:880. doi: 10.1186/s12913-021-06893-4 (PMC8394191; doi:10.1186/s12913-021-06893-4)
Supplement: Supplementary file 2 — Additional file 2:. Theoretical Domains Framework Survey Questions. [file 12913_2021_6893_MOESM2_ESM.docx]

**Additional file 2.** Theoretical Domains Framework Survey Questions

***Please reflect on the following statements and questions about having behaviour change conversations with clients/individuals. The statements and questions have been carefully devised in order to provide a validated evaluation of the Healthy Conversation Skills Training. While many may seem repetitious, the subtle differences are an important part of the evaluation.*** Please circle a response between 1 (strongly disagree) to 7 (strongly agree).

*Note: a ‘behaviour change conversation’ is a talk or discussion with a client/individual about changing their actions or habits to improve their health and wellbeing. It could include topics such as healthy eating, physical activity, maintaining a healthy weight, smoking management, reducing alcohol intake, and mental and emotional health.*

|  | ***Strongly disagree*** | ***Disagree*** | ***Slightly Disagree*** | ***Neutral*** | ***Slightly Agree*** | ***Agree*** | ***Strongly Agree*** |
| --- | --- | --- | --- | --- | --- | --- | --- |
| 1. I have **been trained** **how to** have behaviour change conversations in routine consultations with individuals/clients. | 1 | 2 | 3 | 4 | 5 | 6 | 7 |
| 1. I have **the skills** to have behaviour change conversations in routine consultations with individuals/clients. | 1 | 2 | 3 | 4 | 5 | 6 | 7 |
| 1. I have **practiced** having behaviour change conversations in routine consultations with individuals/clients. | 1 | 2 | 3 | 4 | 5 | 6 | 7 |
| 1. Having behaviour change conversations in routine consultations with individuals/clients is **part of my work** as a health professional. | 1 | 2 | 3 | 4 | 5 | 6 | 7 |
| 1. As a health professional, it is **my job** to have behaviour change conversations in routine consultations with individuals/clients. | 1 | 2 | 3 | 4 | 5 | 6 | 7 |
| 1. It is **my responsibility** as a health professional to have behaviour change conversations in routine consultations with individuals/clients. | 1 | 2 | 3 | 4 | 5 | 6 | 7 |
| 1. Doing behaviour change conversations in routine consultations with individuals/clients is **consistent with my health profession**. | 1 | 2 | 3 | 4 | 5 | 6 | 7 |
| 1. I am confident that I can have behaviour change conversations in routine consultations with individuals/clients **even when individuals/clients are not motivated.** | 1 | 2 | 3 | 4 | 5 | 6 | 7 |
| 1. I am confident that I can have behaviour change conversations in routine consultations with individuals/clients **even when there is little time.** | 1 | 2 | 3 | 4 | 5 | 6 | 7 |
| 1. I am **confident that if I wanted** I could have behaviour change conversations in routine consultations with individuals/clients. | 1 | 2 | 3 | 4 | 5 | 6 | 7 |
| 1. If I have behaviour change conversations in routine consultations with individuals/clients **it will benefit public health.** | 1 | 2 | 3 | 4 | 5 | 6 | 7 |
| 1. If I have behaviour change conversations in routine consultations with individuals/clients **it will have disadvantages for my relationship with individuals/clients.** | 1 | 2 | 3 | 4 | 5 | 6 | 7 |
| 1. I **will definitely** have behaviour change conversations in consultations with individuals/clients in the next 3 months. | 1 | 2 | 3 | 4 | 5 | 6 | 7 |
| 1. I **intend to** have behaviour change conversations in consultations with individuals/clients in the next 3 months. | 1 | 2 | 3 | 4 | 5 | 6 | 7 |
| 1. I have a **clear plan of how I will** have behaviour change conversations in routine consultations with individuals/ clients. | 1 | 2 | 3 | 4 | 5 | 6 | 7 |
| 1. I have a cl**ear plan how often I will** have behaviour change conversations in routine consultations with individuals/clients. | 1 | 2 | 3 | 4 | 5 | 6 | 7 |
| 1. When I need to concentrate to have behaviour change conversations in routine consultations with individuals/clients, I **have no trouble focusing my attention**. | 1 | 2 | 3 | 4 | 5 | 6 | 7 |
| 1. When trying to focus my attention on having behaviour change conversations in routine consultations with individuals/clients, I **have difficulty blocking out distracting thoughts.** | 1 | 2 | 3 | 4 | 5 | 6 | 7 |
| 1. When concentrating on having behaviour change conversations in routine consultations with individuals/clients, I **can focus my attention** so that I become unaware of what’s going on around me. | 1 | 2 | 3 | 4 | 5 | 6 | 7 |
| 1. Behaviour change conversations in routine consultations with individuals/clients is **something I do without thinking.** | 1 | 2 | 3 | 4 | 5 | 6 | 7 |
| 1. I **keep track** of my overall progress towards having behaviour change conversations in routine consultations with individuals/clients. | 1 | 2 | 3 | 4 | 5 | 6 | 7 |
| 1. I am **aware of my day-to-day behaviour** as I work towards having behaviour change conversations in routine consultations with individuals/clients. | 1 | 2 | 3 | 4 | 5 | 6 | 7 |

1. For how many of your **next 10 individuals/clients** do you intend to have behaviour change conversations in consultations? (please circle the number)

| 1 | 2 | 3 | 4 | 5 | 6 | 7 | 8 | 9 | 10 |
| --- | --- | --- | --- | --- | --- | --- | --- | --- | --- |

1. How **strong is your intention** to have behaviour change conversations with individuals/clients in consultations in the next 3 months? (please circle the number)

| ***Not strong at all*** | ***Not so strong*** | ***Slightly strong*** | ***Neutral*** | ***Somewhat strong*** | ***Moderately strong*** | ***Very strong*** |
| --- | --- | --- | --- | --- | --- | --- |
| 1 | 2 | 3 | 4 | 5 | 6 | 7 |

1. Generally, in routine consultations with individuals/clients, how often is **covering something else on your agenda a higher priority** than having behaviour change conversations? (please circle the number)

| ***Never*** | ***Very rarely*** | ***Rarely*** | ***Sometimes*** | ***Occasionally*** | ***Very frequently*** | ***Always*** |
| --- | --- | --- | --- | --- | --- | --- |
| 1 | 2 | 3 | 4 | 5 | 6 | 7 |

1. How often do you **forget** to have behaviour change conversations in routine consultations with individuals/clients? (please circle the number)

| ***Never*** | ***Very rarely*** | ***Rarely*** | ***Sometimes*** | ***Occasionally*** | ***Very frequently*** | ***Always*** |
| --- | --- | --- | --- | --- | --- | --- |
| 1 | 2 | 3 | 4 | 5 | 6 | 7 |
