# Supplementary material for: The impact of Healthy Conversation Skills training on health professionals’ barriers to having behaviour change conversations: a pre-post survey using the Theoretical Domains Framework
Source: BMC Health Serv Res. 2021 Aug 27;21:880. doi: 10.1186/s12913-021-06893-4 (PMC8394191; doi:10.1186/s12913-021-06893-4)
Supplement: Supplementary file 4 — Additional file 4:. Healthy Conversation Skill confidence, importance, usefulness and competence questions. [file 12913_2021_6893_MOESM4_ESM.docx]

**Additional file 4.** Healthy Conversation Skills confidence, importance, usefulness and competence questions

***The following questions ask about your confidence, perceived importance and usefulness of having behaviour change conversations.*** Please circle one number for each item.

1. On a scale of 1 – 10, how **confident** do you feel about supporting clients/individuals to make behaviour changes? (please circle the number)

Not confident Very confident

| 1 | 2 | 3 | 4 | 5 | 6 | 7 | 8 | 9 | 10 |
| --- | --- | --- | --- | --- | --- | --- | --- | --- | --- |

1. On a scale of 1 – 10, how **confident** do you feel about supporting Aboriginal clients/individuals to make behaviour changes? (please circle the number)

Not confident Very confident

| 1 | 2 | 3 | 4 | 5 | 6 | 7 | 8 | 9 | 10 |
| --- | --- | --- | --- | --- | --- | --- | --- | --- | --- |

1. On a scale of 1 – 10, how **important** is it for you to support clients/individuals to make a behaviour change? (please circle the number)

Not important Very important

| 1 | 2 | 3 | 4 | 5 | 6 | 7 | 8 | 9 | 10 |
| --- | --- | --- | --- | --- | --- | --- | --- | --- | --- |

1. On a scale of 1 – 10, how **useful** do you think the conversations you have are at supporting individuals to make a behaviour change? (please circle the number)

Not useful Very useful

| 1 | 2 | 3 | 4 | 5 | 6 | 7 | 8 | 9 | 10 |
| --- | --- | --- | --- | --- | --- | --- | --- | --- | --- |

1. ***Below are four things individuals might say. Please write in the boxes below, the next thing you might say to support this client/individual to make a behaviour change.***

| - 1. *“I need to lose weight, but I don’t like vegetables.”* |
| --- |
| *You say:* |
| - 1. *“I should cut down on my alcohol intake, but my partner likes to open a bottle of wine after work.”* |
| *You say:* |
| - 1. *“I’ve lost count of the number of times I’ve tried to stop smoking—it’s hopeless!”* |
| *You say:* |
| - 1. *“I just don’t seem to have time to do any exercise.”* |
| *You say:* |
