# Supplementary material for: The impact of Healthy Conversation Skills training on health professionals’ barriers to having behaviour change conversations: a pre-post survey using the Theoretical Domains Framework
Source: BMC Health Serv Res. 2021 Aug 27;21:880. doi: 10.1186/s12913-021-06893-4 (PMC8394191; doi:10.1186/s12913-021-06893-4)
Supplement: Supplementary file 5 — Additional file 5:. Acceptability, appropriateness and feasibility questions. [file 12913_2021_6893_MOESM5_ESM.docx]

**Additional file 5.** Survey questions to assess the acceptability, appropriateness and feasibility of using HCS in their role.

***Please reflect on the following statements about using Healthy Conversation Skills in your role.*** Circle one response between 1 (completely disagree) to 5 (completely agree).

|  | | Completely disagree | Disagree | Neither agree nor disagree | Agree | Completely agree |
| --- | --- | --- | --- | --- | --- | --- |
| Using Healthy Conversation Skills…. | 1. …meets my approval | 1 | 2 | 3 | 4 | 5 |
|  | 1. …is appealing to me | 1 | 2 | 3 | 4 | 5 |
|  | 1. …seems fitting | 1 | 2 | 3 | 4 | 5 |
|  | 1. …seems suitable | 1 | 2 | 3 | 4 | 5 |
|  | 1. …seems applicable | 1 | 2 | 3 | 4 | 5 |
|  | 1. …seems like a good match | 1 | 2 | 3 | 4 | 5 |
|  | 1. …seems implementable | 1 | 2 | 3 | 4 | 5 |
|  | 1. …seems possible | 1 | 2 | 3 | 4 | 5 |
|  | 1. …seems doable | 1 | 2 | 3 | 4 | 5 |
|  | 1. …seems easy to use | 1 | 2 | 3 | 4 | 5 |
| 1. I like Healthy Conversation Skills | | 1 | 2 | 3 | 4 | 5 |
| 1. I welcome Healthy Conversation Skills | | 1 | 2 | 3 | 4 | 5 |
