# Supplementary material for: The impact of Healthy Conversation Skills training on health professionals’ barriers to having behaviour change conversations: a pre-post survey using the Theoretical Domains Framework
Source: BMC Health Serv Res. 2021 Aug 27;21:880. doi: 10.1186/s12913-021-06893-4 (PMC8394191; doi:10.1186/s12913-021-06893-4)
Supplement: Supplementary file 6 — Additional file 6:. Healthy Conversation Skills follow-up telephone intervention protocol. [file 12913_2021_6893_MOESM6_ESM.docx]

**Additional file 6.** HCS Follow-up telephone interview protocol

**Follow-up Telephone Interview Evaluation**

*Semi-structured telephone interview protocol*

*Purpose*

Every person who attends the Healthy Conversation Skills training will receive a follow-up telephone call with a member of the training team, 6-10 weeks after training session 2. The purpose of the call is two-fold:

1. To support the implementation of new skills into practice. Simply talking about the skills they learnt, opportunities to use them, and thinking about how to get round any barriers can help embed new practice and encourage trainees to use the skills at every opportunity.

2. To collect evaluation data about changes people have made to their conversations with individuals, since attending the Healthy Conversation Skills training, and assess against the Healthy Conversation Skills competencies rating rubric.

*Set up*

1. Trainees are introduced to the idea of follow-up in the 2nd session. At the end of this session, they arrange a date and a time slot with the facilitators for their follow-up phone call. They are made aware of its purpose and that (with their permission) it will be recorded for evaluation purposes. This enables all calls to be transcribed and coded for evaluation.

2. The dates and times are then entered into a dataset.

*Contacting people*

1. Where contact is made on the phone with a trainee, follow the steps which start below with the ‘to-do-list’.

3. Where contact has not been possible, leave a message or ask for the trainee to return your call. After calling several times with no success, on the final attempt leave a message saying you won’t try contacting them again for now, leave your contact details and encourage the person to get in touch when they can.

4. Where contact has been made, but the follow-up call has not been possible, arrange an appropriate time and day to call. When an appointment has been made, start the call with an assumption that the person has time. Let the person tell you if they need to reschedule, otherwise go ahead with the evaluation.

*To-do-List*

**Before phone call:**

- - Print off or look up contact details for the trainee you are contacting.
  - Check recording equipment is working.
  - Print off phone call follow-up evaluation form – 1 per person.
  - Call each person at the pre-arranged time. Do the phone call there and then if person is willing.
  - Re-call or email those you don’t reach.
  - Remember to reaffirm consent to answer the questions and to gain verbal consent for recording the call.

**After Phone call:**

- Check call has recorded OK.
  - Complete any written notes and reflections from the call.
- Debrief with a team member if necessary—could be to discuss any challenges, talk about what went well / exciting stories or to get support for any difficult/emotional conversations.
  - Update database with trainee details if any changes or additional information.
  - File the phone call follow-up evaluation form in a locked cabinet.
  - Arrange for conversation recording to be transcribed.
  - Arrange for transcripts to be double-coded—use the competencies rubric to rate evidence of using the competencies on a 0-4 scale. Coding sheet and rubric are in the manual. Refer to notes on the conversation where necessary during the coding process.
  - Arrange for scores to be data-entered.

**Semi-structure telephone interview script**

***Get consent for the conversation to be recorded:***

This is for evaluation purposes and so that we can make sure we have similar conversations with everyone who went on the training. Any quotes we use will be anonymous.

Consent given to record: Yes / No

# *Spiel:*

# I’d like you to think back to the Healthy Conversation Skills training and the skills you heard about. Remember we talked about Open Discovery Questions beginning with how and what, and how to incorporate these into conversations with individuals? We hope that you found the training useful and would like to know a bit more about how you are using the skills from the training. Since we saw you last hopefully you’ve had a chance to talk with individuals about their health behaviours, or changing their behaviour in some way. Can you think of an example of any conversations you’ve had? Please tell me the story of how it went? Try to be as specific as possible.

# *Tell the story…*

Questions to use if needed - remember you are assessing the trainee against the competencies rating rubric, familiarise yourself with this rubric so questions you ask address these competencies in order to score them.

- How did the conversation start?
- What was the first thing you said? What else did you say? (ODQ)
- How long did it last?
- How did you feel it went?
- What worked well?
- Who did most of the talking in the conversation?
- How did you help this person plan for change?
- How did you help them set SMARTER goals?

# What has been the value of the “Having a Healthy Conversation Reflection Tool”? How many have you completed?

# How useful did you find the training? What was of value or not of value?

# How do you think you have you used the skills you learnt on the training? What do you think you could do differently?

- What gets in the way of using your Healthy Conversation Skills? What is more important in your day-to-day work with clients?

# How have your conversations changed since attending the training?

We have no more questions. But I would like to thank you for answering these questions for us, we appreciate the time you have spent on this evaluation.

**Coding matrix for responses in telephone interview**

**TO BE COMPLETED INDEPENDENTLY BY 1 or 2 CODERS AFTER THE CONVERSATION** Write example line numbers in the spaces below. Rate success from 0-4 — refer to the Competencies Rating Rubric (see next page). Once completed, arrange to double-code and agree total score.

ID No:

| 1. **Asked Open Discovery Questions** | |
| --- | --- |
| E.g.    Rating: 0 1 2 3 4 | |
| 1. **Reflected on own practice** | |
| E.g.    Rating: 0 1 2 3 4 | |
| 1. **More time spent listening than giving information** | |
| E.g.    Rating: 0 1 2 3 4 | |
| 1. **Supported SMARTER goal-setting** | |
| E.g.    Rating: 0 1 2 3 4 | |
| **Coding comments/ Quotes about training/HCS use:** | **Double-coding**  **agreed total**  **(out of 16):** |

**Competencies rating rubric for telephone interview (0 = worst to 4 = best)**

0 = No demonstration of Healthy Conversation Skills competency

1 = Some demonstration of Healthy Conversation Skills competency

2 = Moderate demonstration of Healthy Conversation Skills competency

3 = Good demonstration of Healthy Conversation Skills competency

4 = Strong demonstration of Healthy Conversation Skills competency

**1 Asked Open Discovery Questions**

**0** No evidence of using Open Discovery Questions at all in conversations

**1** Evidence of an awareness of the difference between Open Discovery Questions & other types of questions, though has not yet used Open Discovery Questions

**2 Limited** evidence of asking Open Discovery Questions, but **not** exploring context

**3 Some** evidence of asking Open Discovery Questions to explore context or plan change

**4 Substantial** evidence of asking Open Discovery Questions to explore context or plan change

**2 Reflected on own practice**

***(Reflection = describes own practice & provides a rationale for, or impact of, that practice)***

**0** No evidence of reflecting on own practice

**1** Evidence of reflecting on own practice, but doesn’t include changes since the training

**2 Limited** evidence of reflecting on own practice, e.g. can say what they do well since the training

**3 Some** evidence of reflecting on own practice, e.g. can say what they do well & not so well since the training

**4 Substantial** evidence of reflecting on own practice, e.g. can clearly articulate their strengths & identify areas for improvement in using the skills from the course

**3 More time spent listening than giving information**

***(When there’s a lack of explicit evidence for either of these, look at the bigger picture)***

**0** Evidence they spend the whole conversation giving information rather than listening

**1** Evidence they spend more time in conversations giving information than listening

**2** Evidence they spend equal amount of time in conversations giving information as listening

**3** Evidence they spend a little more time in conversations listening than giving information

**4** Evidence they spend substantially more time in conversations listening than giving information

**4 Supported SMARTER goal-setting**

**0** No evidence of supporting planning

**1** No evidence of supporting planning, but evidence that they would like to

**2** Evidence of supporting planning by asking questions but not using the SMARTER technique **OR**

Evidence of using **SMARTER** techniques, but not encouraging people to make their own plans

**3** Evidence that they support **SMARTER** planning by asking questions to encourage people to come up with their own plans

**4** Evidence that they support **SMARTER** planning by asking questions to encourage people to come up with their own plans & has followed-up / intends to follow-up on those plans
