# Supplementary material for: The impact of Healthy Conversation Skills training on health professionals’ barriers to having behaviour change conversations: a pre-post survey using the Theoretical Domains Framework
Source: BMC Health Serv Res. 2021 Aug 27;21:880. doi: 10.1186/s12913-021-06893-4 (PMC8394191; doi:10.1186/s12913-021-06893-4)
Supplement: Supplementary file 7 — Additional file 7:. Moderate, high and very high correlations of the pre and post Theoretical Domains Framework domains in participants from the Healthy Conversation Skills training. [file 12913_2021_6893_MOESM7_ESM.docx]

**Additional file 7.** Moderate, high and very high correlations of the pre- and post-training TDF domains in participants undertaking HCS training (n=63) (p<0.05).

|  | Skills | Social/ professional role and identity | Beliefs about capabilities | Beliefs about consequences | Intentions | Goals | Memory, attention and decision processes | Behaviour Regulation |
| --- | --- | --- | --- | --- | --- | --- | --- | --- |
| **Pre Training** |  |  |  |  |  |  |  |  |
| Skills | - |  |  |  |  |  |  |  |
| Social/professional role and identity | 0.50 | - |  |  |  |  |  |  |
| Beliefs about capabilities | 0.68 | 0.52 | - |  |  |  |  |  |
| Beliefs about consequences |  |  |  | - |  |  |  |  |
| Intentions |  | 0.58 |  |  | - |  |  |  |
| Goals | 0.57 |  | 0.57 |  |  | - |  |  |
| Memory, attention and decision processes |  |  |  |  | 0.52 |  | - |  |
| Behavioural regulation |  |  | 0.52 |  |  |  |  | - |
| **Post Training** |  |  |  |  |  |  |  |  |
| Skills | - |  |  |  |  |  |  |  |
| Social/professional role and identity | 0.62 | - |  |  |  |  |  |  |
| Beliefs about capabilities | 0.54 |  | - |  |  |  |  |  |
| Beliefs about consequences |  | 0.65 |  | - |  |  |  |  |
| Intentions |  | 0.53 | 0.59 |  | - |  |  |  |
| Goals |  |  | 0.59 |  |  | - |  |  |
| Memory, attention and decision processes |  |  |  |  |  |  | - |  |
| Behavioural regulation | 0.54 |  |  |  |  |  |  | - |
