# Supplementary material for: The impact of Healthy Conversation Skills training on health professionals’ barriers to having behaviour change conversations: a pre-post survey using the Theoretical Domains Framework
Source: BMC Health Serv Res. 2021 Aug 27;21:880. doi: 10.1186/s12913-021-06893-4 (PMC8394191; doi:10.1186/s12913-021-06893-4)
Supplement: Supplementary file 8 — Additional file 8:. The impact of HCS training on practice as reported by the types of response provided by participants. [file 12913_2021_6893_MOESM8_ESM.docx]

**Additional file 8:** The impact of HCS training on practice as reported by the types of response provided by participants to four written statements about behaviour change immediately before (n=64) and after training (n=62), and at 3 months follow-up (n=34).

| Response styles | **Pre-training** | | | **Post-training** | | | **Follow-up** | | |
| --- | --- | --- | --- | --- | --- | --- | --- | --- | --- |
|  | **Clinical**  **(n=37)** | **Non-clinical**  **(n=27)** | **Total participants**  **(n=64)** | **Clinical**  **(n=36)** | **Non-clinical**  **(n=26)** | **Total participants**  **(n=62)** | **Clinical**  **(n=17)** | **Non-clinical**  **(n=17)** | **Total participants**  **(n=34)** |
|  | **N (%)** | **N (%)** | **N (%)** | **N (%)** | **N (%)** | **N (%)** | **N (%)** | **N (%)** | **N (%)** |
| Telling/suggesting | 60 (41%) | 35 (32%) | 95 (37%) | 1 (1%) | 0 (0%) | 1 (1%) | 0 (0%) | 1 (1%) | 1 (1%) |
| In my experience | 4 (3%) | 6 (5%) | 10 (4%) | 0 (0%) | 0 (0%) | 0 (0%) | 0 (0%) | 0 (0%) | 0 (0%) |
| Reflection/empathy | 18 (12%) | 12 (11%) | 20 (12%) | 0 (0%) | 0 (0%) | 0 (0%) | 4 (6%) | 0 (0%) | 4 (3%) |
| Closed question | 24 (16%) | 22 (20%) | 46 (18%) | 0 (0%) | 0 (0%) | 0 (0%) | 0 (0%) | 1 (1%) | 1 (1%) |
| Open other question | 2 (1%) | 2 (2%) | 4 (2%) | 2 (1%) | 5 (4%) | 7 (2%) | 0 (0%) | 4 (6%) | 4 (3%) |
| Open discovery question (1^st^ or 2^nd^ response) | 32 (22%) | 31 (29%) | 63 (25%) | 141 (98%) | 98 (94%) | 239 (96%) | 56 (82%) | 62 (91%) | 118 (87%) |
| Missing response | 8 (5%) | 0 (0%) | 8 (3%) | 0 (0%) | 1 (2%) | 1 (2%) | 8 (12%) | 0 (0%) | 8 (6%) |
| Total responses | 148 (100%) | 108 (100%) | 256 (100%) | 144 (100%) | 104 (100%) | 248 (100%) | 68 (100%) | 68 (100%) | 136 (100%) |
